# Factors Associated With Protective Mask-Wearing Behavior to Avoid COVID-19 Infection in China: Internet-Based Cross-sectional Study

**DOI:** 10.2196/32278

**Published:** 2022-05-26

**Authors:** Yue Xu, Qingqing Wu, Shuiyang Xu, Yusui Zhao, Xuehai Zhang

**Affiliations:** 1 Department of Health Education Zhejiang Provincial Center for Disease Control and Prevention Hangzhou China

**Keywords:** COVID-19, internet-based, disease prevention, mask, knowledge, behavior

## Abstract

**Background:**

The novel coronavirus disease COVID-19 is likely to spread from person to person in close-contact settings. The Chinese Center for Disease Control and Prevention released a handbook on COVID-19, which introduced health information to the public, specifically related to wearing masks correctly and adopting preventive measures to avoid COVID-19 infection.

**Objective:**

The aim of this study was to assess the level of mask knowledge, behavior related to mask usage, and major information channels used for obtaining mask- and COVID-19–related information in China.

**Methods:**

An internet-based survey was conducted primarily using DingXiang Doctor WeChat public accounts. The data about mask knowledge and behavior were collected and analyzed. In addition to descriptive statistics, logistic regression was used to analyze significant risk factors contributing to protective mask behavior.

**Results:**

Data were collected from a total of 10,304 respondents to the survey. More than half of the respondents were under 30 years old and nearly three-quarters were women. Over 80% of participants had a bachelor’s degree or higher, and the largest proportion of respondents (n=4204, 40.80%) were employed as business/service workers. Over half of the study participants were married (n=5302, 51.46%). The findings revealed that 67.49% (6954/10,304) of the participants practiced protective mask behavior; 97.93% (10,091/10,304) believed that wearing masks is an effective protective measure against COVID-19; 96.85% (9979/10,304) chose a mask that has two or more layers of washable, breathable fabric; and 70.57% (7272/10,304) wore the masks correctly. Gender, age, occupation, and education level had significant effects on behavior, whereas marital status and the infection status of family members were not significantly related to mask-wearing behavior. In addition, WeChat public accounts (9227/10,304, 89.55%) were the most prominent source of obtaining health information for Chinese netizens after the outbreak of COVID-19.

**Conclusions:**

This study elucidated that Chinese netizens’ protective mask behavior is far lower than their mask-related knowledge. Improved information channels and adequate information on wearing masks are necessary to improve the public’s protective mask behavior, particularly among men, the elderly, and people with less education.

## Introduction

The COVID-19 pandemic is considered a global public health emergency of serious concern [1-3]. The disease is caused by the novel coronavirus SARS-CoV-2. Due to its rapid spread, extremely harmful effects, and pathogenic complexity, the World Health Organization escalated the risk assessment of COVID-19 to “very high” [4]. By December 1, 2020, there were 44,579,298 cases and 1,494,630 deaths due to COVID-19 confirmed across the world [5].

The COVID-19 virus is highly infectious, which mainly spreads from person to person through respiratory droplets [4]. At the time of submission of this paper, target-specific drugs and vaccines were not yet available for protection against COVID-19. Hence, controlling the outbreak and taking proper measures to protect people became crucial. Among the control measures implemented, face masking has been shown to mitigate the transmission of SARS-CoV-2 by creating a physical barrier, making it one of the most efficient measures to prevent COVID-19 [6-8]. Although studies have found differences in the protective effect of different types of masks [7,8], the modeling predictions suggest that even the use of relatively ineffective masks can decrease community transmission of the virus relative to no masks [9]. China has taken the toughest measures to require the public to wear masks in public since the beginning of the outbreak in early 2020, mainly by restricting access to public places such as hospitals and shopping malls or prohibiting travel on public transport for nonmask-wearers. Even in a phase when the outbreak is gradually under control, face masks have become the new default social norm for the Chinese public.

From January 20, 2020, when person-to-person transmission was confirmed and widely known by the public [10], a wide-ranging, multilevel health education campaign against COVID-19 was carried out in China. Many promotional materials related to COVID-19 were compiled by health experts in China, such as the COVID-19 Guidelines for Public Protection (version 2) [11] and the guidebook on COVID-19 prevention [12]. The dissemination of the core content of these guidelines through the internet was perceived to be highly effective in setting up the desired health behavior and lifestyle to control the COVID-19 pandemic [13,14].

There are many major communication channels used to spread information on COVID-19 in China, such as WeChat, microblogs, television, radio, and other media outlets. Owing to the increased global access to the internet over the past decade, people have been more willing to acquire relevant knowledge over the internet [15] compared with the situation during the severe acute respiratory syndrome (SARS) outbreak in 2003 [16]. WeChat has grown into the largest and most influential social network in China, with 963 million active users [17]. It is possible for WeChat users to subscribe to the customer service from the WeChat public accounts and obtain specific information they desire. *DingXiang Doctor* is the most influential professional WeChat public account in the health field across China [18,19], which is effectively used to disseminate health information to the general population. Previous research in this field [20] has addressed the issue of Chinese netizens’ effective access to desired COVID-19 information; however, there is a lack of further in-depth study on specific protective behavior. Therefore, the aim of this study was to describe Chinese netizens’ behaviors related to wearing a mask and their relationship with internet content on mask-related information. We obtained representative data to assess the popularity of wearing masks through an in-depth analysis of data from an internet-based cross-sectional survey.

## Methods

### Participants

An internet-based cross-sectional survey was conducted from January 31 to February 2, 2020, at the beginning of the COVID-19 epidemic in China. A message stating “COVID-19, have you done enough to prevent it?” was created online with a link to the questionnaire. The target population for the survey was defined as all residents aged 15 years and above living in China. Participation in the study was purely voluntary, without any financial incentive.

### Data Collection

The research tool used in the study was designed by health education experts from the Center for Disease Control and Prevention of Zhejiang Province. The survey contained four segments of 15 multiple-choice questions, including sociodemographic information (eg, gender, age, occupation, education level, marital status), infection status, COVID-19–related knowledge, and mastery of preventive measures (eg, mask wearing, hand washing). The questionnaire has been validated, demonstrating good reliability and validity [20]. The data were gathered using *DingXiang Doctor* WeChat public accounts.

### Measures

#### Protective Mask Behavior

**Figure 1:**
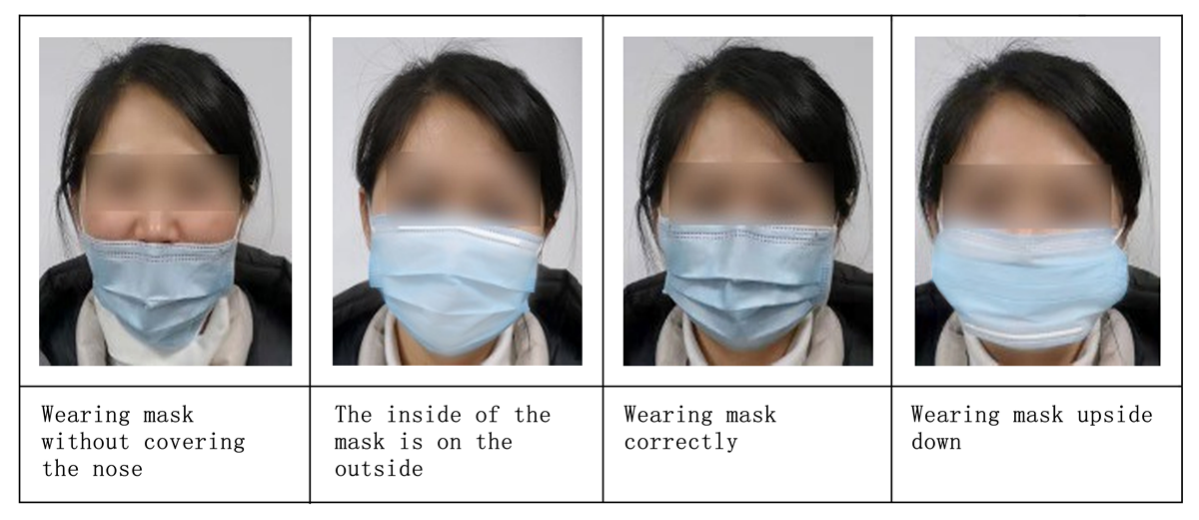
Images of the four ways of wearing masks used in the study.

Protective mask behavior was set as the dependent variable in this analysis, which was measured by asking the respondents on their perceptions of the protective effects of masks, their choice of mask type, and the way they wore the mask. Respondents were considered to be performing “protective mask behavior” if the responses to all three questions matched the statements: (1) “I believe that wearing a mask is effective to protect against COVID-19,” (2) “I choose to wear a medical mask (two or more layers of washable, breathable fabric),” and (3) “I usually wear my mask as shown in the third image from the left” (Figure 1).

Relevant independent variables included in the analysis were obtained through self-report, comprising gender (male, female), age (15-20 years, 21-30 years, 31-40 years, 41-50 years, 51-60 years, 61 years or older), education level (primary or below, secondary, undergraduate, postgraduate or above), occupation (government institution staff, business/service worker, student, medical staff, homemaker, retired/unemployed), marital status (single/divorced/widowed, married), and COVID-19 infection status of family members (confirmed case/suspected case, close contact with a confirmed case, none of the above).

#### Knowledge About COVID-19 Messages

The information channels that the participants used were assessed by asking the following question: “during the past 30 days, have you seen or received messages related to COVID-19 via the following channels?: (1) friends/relatives/colleagues, (2) websites, (3) WeChat public account, (4) microblogs, (5) WeChat, (6) news apps, (7) television/radio, (8) newspapers, (9) SMS text messages, (10) community outreach.” The response options included “yes” and “no” to each category.

### Patient and Public Involvement

The analyses were based on existing data of an internet-based cross-sectional survey. To our knowledge, no participants were involved in the design, recruitment, or conduct of the study. The research question and outcome measures of the study were determined by factors reported to be associated with protective mask behavior [21,22]. Thus, we could not disseminate the results to each participant; however, the results will be disseminated to the public through broadcasts and popular science articles.

### Ethics

This study was approved by the Ethics Committee of the Zhejiang Provincial Center for Disease Control and Prevention (approval number: 2020-009). Informed consent was obtained from all participants before collecting their information. To protect the participants’ confidentiality, we kept all data confidential and without any identifiers.

### Statistical Analysis

SPSS version 18.0 was used for all analyses. Standard descriptive statistics were used to summarize demographic variables and other parameters that might be associated with protective mask behavior. Logistic regression was applied to determine the factors associated with protective mask behavior based on survey and self-report data. Two-sided *P* values <.05 were considered statistically significant.

## Results

### General Participant Characteristics

During the study period, a total of 590,000 *DingXiang Doctor* users visited the online page, 10,304 of whom responded to the survey. The sociodemographic characteristics are summarized in Table 1. Overall, the majority of respondents were women and slightly more than half were married. The age group with the highest proportion was 21-30 years, followed by 31-40 years, 41-50 years, 15-20 years, 51-60 years, and ≥60 years. Most of the respondents had an undergraduate degree, followed by postgraduate or above, secondary, and primary education or below. The majority of the respondents were employed as business/service workers, followed by medical staff, government institution staff, retired/unemployed, students, and homemakers. Less than 1% of the respondents had a confirmed/suspected case of COVID-19 in the family, and approximately 4% had close contact with a confirmed case (Table 1).

**Table 1:** Survey respondents’ sociodemographic characteristics (N=10,304).

Sociodemographic characteristics			Respondents, n (%)
**Gender**			
	Male	2670 (25.91)	
	Female	7634 (74.09)	
**Age (years)**			
	15-20	901 (8.74)	
	21-30	4830 (46.88)	
	31-40	2945 (28.58)	
	41-50	1141 (11.07)	
	51-60	403 (3.91)	
	>61	84 (0.82)	
**Education level**			
	Primary or below	526 (5.10)	
	Secondary	1117 (10.84)	
	Undergraduate	7219 (70.06)	
	Postgraduate or above	1442 (13.99)	
**Occupation**			
	Government institution staff	1729 (16.78)	
	Business/service worker	4204 (40.80)	
	Students	668 (6.48)	
	Medical staff	1894 (18.38)	
	Homemaker	202 (1.96)	
	Retired/unemployed	1607 (15.60)	
**Marital status**			
	Single/divorced/widowed	5002 (48.54)	
	Married	5302 (51.46)	
**COVID-19 infection status of family members**			
	Confirmed case/suspected case	70 (0.68)	
	Close contact with a confirmed case	360 (3.49)	
	None of the above	9874 (95.83)	

### Protective Mask Behavior

Table 2 shows the level of protective mask behavior by various sociodemographic factors. For instance, the majority (>65%) of the participants practiced protective mask behavior, and the great majority (>95%) believed that wearing a mask is effective protection against COVID-19 and chose a mask that has two or more layers of washable, breathable fabric. In addition, over 70% wore masks correctly (as shown in Figure 1). The percentage of practicing protective mask behavior varied with differences in gender, age, education, occupation, marital status, and infection status of family members. It was higher among women than men. Older respondents tended to have a lower level of protective mask behavior; even among the oldest age group (61 years or older), over 50% practiced this behavior. Education level was positively associated with protective mask behavior. Even among respondents with a primary education level or below, the majority practiced protective mask behavior. The highest protective behavior level was found in the business/service worker group, married group, and those with a positive infection status of family members relative to other corresponding categories.

**Table 2:** Distribution of respondents who practice protective mask behavior according to sociodemographic characteristics.

Sociodemographic characteristic		Believe that wearing a mask is effective protection against COVID-19, n (%)	Choose a mask that has two or more layers of washable, breathable fabric, n (%)	Wearing mask correctly, n (%)	Protective mask behavior^a^, n (%)
**Gender**					
	Male (n=2670)	2592 (97.08)	2578 (96.55)	1764 (66.07)	1663 (62.28)
	Female (n=7634)	7499 (98.23)	7401 (96.95)	5508 (72.15)	5291 (69.31)
**Age (years)**					
	15-20 (n=901)	886 (98.34)	865 (96.00)	629 (69.81)	606 (67.26)
	21-30 (n=4830)	4726 (97.85)	4661 (96.50)	3496 (72.38)	3322 (68.78)
	31-40 (n=2945)	2892 (98.20)	2880 (97.79)	2079 (70.59)	2005 (68.08)
	41-50 (n=1141)	1113 (97.55)	1112 (97.46)	774 (67.84)	742 (65.03)
	51-60 (n=403)	393 (97.52)	383 (95.04)	249 (61.79)	234 (58.06)
	>61 (n=84)	81 (96.43)	78 (92.86)	45 (53.57)	45 (53.57)
**Education level**					
	Primary or below (n=526)	511 (97.15)	505 (96.01)	339 (64.45)	324 (61.60)
	Secondary (n=1117)	1092 (97.76)	1072 (95.97)	788 (70.550	747 (66.88)
	Undergraduate (n=7219)	7069 (97.92)	7000 (96.97)	5121 (70.94)	4895 (67.81)
	Postgraduate or above (n=1442)	1419 (98.40)	1402 (97.23)	1024 (71.01)	988 (68.52)
**Occupation**					
	Government institution staff (n=1729)	1697 (98.15)	1687 (97.57)	1189 (68.77)	1149 (66.45)
	Business/service worker (n=4204)	4109 (97.74)	4088 (97.24)	3037 (72.24)	2895 (68.86)
	Students (n=668)	662 (99.10)	661 (98.95)	444 (66.47)	436 (65.27)
	Medical staff (n=1894)	1852 (97.78)	1810 (95.56)	1350 (71.28)	1281 (67.63)
	Homemaker (n=202)	198 (98.02)	192 (95.05)	131 (64.85)	123 (60.89)
	Retired/unemployed (n=1607)	1573 (97.88)	1541 (95.89)	1121 (69.76)	1070 (66.58)
**Marital status**					
	Single/divorced/widowed (n=5002)	4909 (98.14)	4817 (96.30)	3590 (71.77)	3421 (68.39)
	Married (n=5302)	5182 (97.74)	5162 (97.36)	3682 (69.45)	3533 (66.64)
**COVID-19 infection status of family members**					
	Confirmed case/suspected case (n=70)	68 (97.14)	66 (94.29)	49 (70.00)	46 (65.71)
	Close contact with a confirmed case (n=360)	349 (96.94)	343 (95.28)	263 (73.06)	243 (67.50)
	None of the above (n=9874)	9674 (97.97)	9570 (96.92)	6960 (70.49)	6665 (67.50)
Overall (N=10,304)		10,091 (97.93)	9979 (96.85)	7272 (70.57)	6954 (67.49)

^a^Calculated according to the number of respondents giving correct responses to the three questions.

### Factors Associated With Protective Mask Behavior

A multivariate logistic analysis was performed on six factors (gender, age, education, occupation, marital status, and infection status of family members). Of these factors, gender, age, education, and occupation were significantly associated with the implementation of protective masking behavior, whereas marital status and infection status of family members did not show a significant association with the outcome. The findings showed that males were 73% more likely to have protective mask behavior compared to females. Compared to the ≥60 years age group, the proportion of respondents practicing protective mask behavior was higher in the age groups of 15-20 years, 21-30 years, 31-40 years, and 41-50 years, suggesting that the proportion of mask behavior decreases with age. People with secondary education and below were less likely to engage in protective masking behavior compared to those with higher education. In the occupational category, the business/service workers exhibited better mask behavior compared with the retired/unemployed class (see Table 3).

**Table 3:** Factors associated with protective mask behavior.

Covariate		OR^a^ (95% CI)	*P* value
**Gender**			
	Male	0.73 (0.66-0.88)	<.001
	Female	Reference	—^b^
**Age (years)**			
	15-20	2.03 (1.15-3.59)	.01
	21-30	1.95 (1.14-3.34)	.01
	31-40	1.91 (1.12-3.26)	.02
	41-50	1.76 (1.03-3.02)	.04
	51-60	1.24 (0.74-2.07)	.41
	>61	Reference	—
**Education level**			
	Primary or below	0.77 (0.62-0.97)	.02
	Secondary	0.96 (0.81-1.15)	.69
	Undergraduate	0.97 (0.86-1.10)	.66
	Postgraduate or above	Reference	—
**Occupation**			
	Government institution staff	1.02 (0.88-1.19)	.75
	Business/service worker	1.16 (1.02-1.32)	.02
	Students	0.96 (0.79-1.17)	.69
	Medical staff	1.04 (0.87-1.24)	.70
	Homemaker	1.27 (0.86-1.89)	.23
	Retired/unemployed	Reference	—
**Marital status**			
	Single/divorced/widowed	1.03 (0.91-1.15)	.67
	Married	Reference	—
**Infection status of family members**			
	Confirmed case/suspected case	0.90 (0.55-1.49)	.69
	Close contact with a confirmed case	0.98 (0.78-1.22)	.83
	None of the above	Reference	—

^a^OR: odds ratio.

^b^Not applicable.

### Knowledge of Messages Against COVID-19

There were 10 major channels through which the public had seen messages against COVID-19, including (in descending order of popularity) WeChat public accounts, news apps, WeChat, television/radio, microblogs, friends/relatives/​colleagues, websites, SMS, community outreach, and newspapers (Table 4). The education distribution indicated a step gradient; respondents with higher education used more information channels than others. The student community primarily received information from new media sources such as WeChat rather than through the traditional media such as television. Homemakers were more likely to obtain information from television/radio compared with other occupation groups.

**Table 4:** Information channels through which respondents obtain information about COVID-19.

Sociodemographic characteristics		Friends/ relatives/ colleagues, n (%)	Websites, n (%)	WeChat public account, n (%)	Microblogs, n (%)	WeChat, n (%)	News apps, n (%)	Television/radio, n (%)	Newspapers, n (%)	SMS, n (%)	Community outreach, n (%)
**Gender**											
	Male (n=2670)	689 (25.81)	898 (33.63)	2355 (88.20)	954 (35.73)	1280 (47.94)	1529 (57.27)	1264 (47.34)	257 (9.63)	449 (16.82)	462 (17.30)
	Female (n=7634)	2180 (28.56)	1780 (23.32)	6872 (90.02)	3605 (47.22)	3884 (50.88)	4219 (55.27)	3378 (44.25)	519 (6.80)	1333 (17.46)	1246 (16.32)
**Age (years)**											
	<20 (n=901)	327 (36.29)	256 (28.41)	812 (90.12)	447 (49.61)	362 (40.18)	445 (49.39)	382 (42.40)	69 (7.66)	220 (24.42)	142 (15.76)
	21-30 (n=4830)	1402 (29.03)	1132 (23.44)	4349 (90.04)	2770 (57.35)	2349 (48.63)	2565 (53.11)	2018 (41.78)	331 (6.85)	789 (16.34)	689 (14.27)
	31-40 (n=2945)	745 (25.30)	757 (25.70)	2671 (90.70)	1019 (34.60)	1610 (54.67)	1709 (58.03)	1317 (44.72)	192 (6.52)	441 (14.97)	477 (16.20)
	41-50 (n=1141)	287 (25.15)	384 (33.65)	980 (85.89)	250 (21.91)	619 (54.25)	732 (64.15)	599 (52.50)	122 (10.69)	235 (20.60)	290 (25.42)
	51-60 (n=403)	90 (22.33)	127 (31.51)	343 (85.11)	66 (16.38)	179 (44.42)	250 (62.03)	267 (66.25)	53 (13.15)	81 (20.10)	96 (23.82)
	> 61 (n=84)	18 (21.43)	22 (26.19)	72 (85.71)	7 (8.33)	45 (53.57)	47 (55.95)	59 (70.24)	9 (10.71)	16 (19.05)	14 (16.67)
**Education level**											
	Primary or below (<9 years) (n=526)	168 (31.94)	121 (23.00)	434 (82.51)	120 (22.81)	218 (41.44)	313 (59.51)	252 (47.91)	43 (8.17)	122 (23.19)	104 (19.77)
	Secondary (10-12 years) (n=1117)	318 (28.47)	333 (29.81)	975 (87.29)	400 (35.81)	484 (43.33)	719 (64.37)	528 (47.27)	87 (7.79)	271 (24.26)	227 (20.32)
	Undergraduate (13-16 years) (n=7219)	1970 (27.29)	1866 (25.85)	6488 (89.87)	3423 (47.42)	3649 (50.55)	4028 (55.80)	3248 (44.99)	568 (7.87)	1230 (17.04)	1228 (17.01)
	Postgraduate or above (> 16 years) (n=1442)	413 (28.64)	358 (24.83)	1330 (92.23)	616 (42.72)	813 (56.38)	688 (47.71)	614 (42.58)	78 (5.41)	159 (11.03)	149 (10.33)
**Occupation**											
	Government institution staff (n=1729)	460 (26.60)	503 (29.09)	1583 (91.56)	700 (40.49)	966 (55.87)	1008 (58.30)	812 (46.96)	128 (7.40)	291 (16.83)	307 (17.76)
	Business/service worker (n=4204)	1091 (25.95)	972 (23.12)	3795 (90.27)	1889 (44.93)	2139 (50.88)	2354 (55.99)	1890 (44.96)	283 (6.73)	638 (15.18)	642 (15.27)
	Students (n=668)	226 (33.83)	247 (36.98)	551 (82.49)	238 (35.63)	403 (60.33)	439 (65.72)	328 (49.10)	110 (16.47)	166 (24.85)	193 (28.89)
	Medical staff (n=1894)	617 (32.58)	515 (27.19)	1706 (90.07)	1059 (55.91)	797 (42.08)	871 (45.99)	807 (42.61)	144 (7.60)	376 (19.85)	245 (12.94)
	Homemaker (n=202)	51 (25.25)	55 (27.23)	175 (86.63)	25 (12.38)	95 (47.03)	125 (61.88)	142 (70.30)	23 (11.39)	41 (20.30)	51 (25.25)
	Retired/unemployed (n=1607)	424 (26.38)	386 (24.02)	1417 (88.18)	648 (40.32)	764 (47.54)	951 (59.18)	663 (41.26)	88 (5.48)	270 (16.80)	270 (16.80)
**Marital status**											
	Single/divorced/widowed (n=5002)	1532 (30.63)	1234 (24.67)	4511 (90.18)	2824 (56.46)	2339 (46.76)	2527 (50.52)	2183 (43.64)	354 (7.08)	880 (17.59)	689 (13.77)
	Married (n=5302)	1337 (25.22)	1444 (27.24)	4716 (88.95)	1735 (32.72)	2825 (53.28)	3221 (60.75)	2459 (46.38)	422 (7.96)	902 (17.01)	1019 (19.22)
**COVID-19 infection status of family members**											
	Confirmed case/suspected case (n=70)	22 (31.43)	20 (28.57)	61 (87.14)	35 (50.00)	44 (62.86)	37 (52.86)	25 (35.71)	5 (7.14)	17 (24.29)	10 (14.29)
	Close contact with a confirmed case (n=360)	107 (29.72)	79 (21.94)	328 (91.11)	148 (41.11)	196 (54.44)	181 (50.28)	140 (38.89)	19 (5.28)	60 (16.67)	59 (16.39)
	None of the above (n=9874)	2740 (27.75)	2579 (26.12)	8838 (89.51)	4376 (44.32)	4924 (49.87)	5530 (56.01)	4477 (45.34)	752 (7.62)	1705 (17.27)	1639 (16.60)
Overall (n=10,304)		2869 (27.84)	2678 (25.99)	9227 (89.55)	4559 (44.24)	5164 (50.12)	5748 (55.78)	4642 (45.05)	776 (7.53)	1782 (17.29)	1708 (16.58)

## Discussion

Our study suggested that approximately two-thirds of the sampled population practiced protective mask behavior. The majority of respondents believed that wearing a mask was effective for protecting themselves from COVID-19; however, only approximately 70% of the respondents appeared to be wearing the mask correctly. There were many channels used for people to obtain COVID-19–related information; however, the WeChat public account was the most important channel for the respondents to obtain prevention knowledge about COVID-19.

From the results of this survey, 97.93% of Chinese netizens believed that wearing a mask was an effective protective measure against COVID-19, which was much higher than found in a previous study performed in Shanghai (45.7%) [23]. This phenomenon is possibly because the Chinese government and the relevant departments resorted to a variety of promotional work, disseminating information through various media [24] in early February in China. The information included requirements for wearing masks in a one-sided manner, but did not teach the public to specifically adopt the protective mask behavior. However, the limited depth of health-awareness information promoted to the public may have an effect on changing people’s mask-wearing behavior to help control the COVID-19 pandemic. With the spread of the COVID-19 epidemic, the need for protective mask behavior was promoted through different major information channels [25].

Although we found that 97.93% of the respondents recognized the importance of wearing a mask for epidemic protection, only 67.49% of the Chinese netizens practiced protective mask behavior. In fact, the Chinese government enforces strict epidemic prevention measures, and face masks are required in all public places as well as indoor areas, which results in a very high rate of mask-wearing (99%) among the Chinese population [26]. In daily life, it is common to see masks being pulled to one side or resting on the chin without completely covering the nose and mouth. Obviously, wearing a mask prevents people from eating, communicating, and other regular activities involving the mouth, and also creates an uncomfortable feeling that could disrupt breathing. Nevertheless, there is no doubt that the use of masks remains the most cost-effective intervention to contain the COVID-19 pandemic [6]. Wearing masks incorrectly does not prevent the spread of the virus [27]. Therefore, based on the previous advocacy for people to carry out self-protection, we should strengthen the guidance on the details of protective measures, such as providing instructions for use in the mask packaging and using representative social media such as WeChat to disseminate more detailed videos on mask-wearing. In addition, in real-world settings, behavioral coaching can be carried out by relying on specific groups such as schools and companies, thereby improving the implementation and effectiveness of public health strategies. However, it is necessary to emphasize that the main body of self-protection is still the individual; thus, continuous publicity of epidemic prevention knowledge and health education for the public are still the most basic effective measures.

In addition, we found that protective mask behavior was significantly associated with gender, age, occupation, and education level for all respondents. These results are consistent with the findings obtained during past outbreaks of SARS and H1N1 influenza virus, where age, gender, and education level were also predictors of face mask usage [28-30]. We found that younger and more educated people showed a higher likelihood of practicing protective masking behavior. This could be due to the fact that these individuals usually spend more time online, and are able to understand relevant health information and implement self-protective behavior correctly. However, research has also suggested that the provision of more comprehensive instructions on mask usage is the strongest predictor of better compliance with mask-wearing, regardless of educational background [26]. This reminds us that it is necessary to provide highly accessible behavioral guidance information for all groups of people. In addition, a higher proportion of commercial/service workers practiced protective masking behaviors than the rest of the population, which could be attributed to the fact that these individuals typically require human contact during working hours and thus may be more concerned with self-protection against COVID-19. Gender differences were also evident, with men implementing protective mask behavior at a lower rate than women, which is consistent with the findings of another cross-national sample survey of face mask use [31]. Women are generally more health-conscious, and a previous study found that women are more anxious and worried about outbreaks than men [32], which leads to more effective self-protective behaviors.

In summary, men, the elderly, and people with less education should be the focus groups of health education on protective mask behavior, and all efforts need to be made to improve the protective mask behavior of the entire population.

With the rapid development of the internet, the way the public acquires health information has changed dramatically. This study found that accesses to health information has shifted from mass media such as television/radio and newspapers to the mobile internet, including WeChat public accounts, news apps, and Weibo. However, subgroups with different characteristics had completely different tendencies. Men appear to be more inclined to access information through websites. For students, WeChat and websites were the main information channels. Television/radio and newspapers, as traditional information channels, have less impact than previously [33]; however, the homemakers surveyed in this study still preferred to obtain information through these traditional channels. For the less educated, traditional interpersonal communication is more common. As such, we should take a cue from these findings to deliver more targeted health information to different groups of people. For instance, mobile users are usually younger and can be targeted with more interesting science videos; television/radio channels tend to include more family health information and preventive measures; and the traditional approach of community outreach can be used to raise awareness of protective mask behavior among the less educated or the elderly.

Within the context of the COVID-19 epidemic, this study relied on the internet to conduct the survey, which was user-friendly to implement and more accessible to a large sample size. However, there were a few limitations. First, the survey used data provided by the respondents’ reports, which may be subject to recall bias and social desirability. Mask covering is a particular behavioral norm during the epidemic, and people tend to either intentionally or unintentionally omit or deny their own violations of the norm; thereby, the extent to which Chinese netizens master mask knowledge and behaviors may be overestimated. Second, protective mask behavior is relatively difficult to measure and inconsistently defined across studies; for example, some studies only consider face masks covering the mouth and nose and secured to the chin as good mask-wearing behavior [34]. On this basis, our study added consideration of personal beliefs as well as mask materials, which can reveal protective mask behavior more comprehensively to a certain extent. Nonetheless, the measurement of behavior is complex and further research is needed to improve the precision of the results.

In summary, an internet-based cross-sectional survey was employed to study the protective mask behavior of sampled respondents. The results showed that Chinese netizens’ protective mask behavior was lower than their mask knowledge. Improved information channels and focused message content related to wearing a mask are necessary to improve the public’s protective mask behavior, particularly among men, the elderly, and people with less education.

## Abbreviations

SARS: severe acute respiratory syndrome
